# Dynamic imaging of adaptive stress response pathway activation for prediction of drug induced liver injury

**DOI:** 10.1007/s00204-018-2178-z

**Published:** 2018-03-03

**Authors:** Steven Wink, Steven W. Hiemstra, Suzanne Huppelschoten, Janna E. Klip, Bob van de Water

**Affiliations:** Division of Drug Discovery and Safety, Leiden Academic Centre for Drug Research, Leiden University, Einsteinweg 55, 2333 CC Leiden, The Netherlands

**Keywords:** High content imaging, Live cell imaging, Adaptive stress pathways, DILI, DILI prediction

## Abstract

Drug-induced liver injury remains a concern during drug treatment and development. There is an urgent need for improved mechanistic understanding and prediction of DILI liabilities using in vitro approaches. We have established and characterized a panel of liver cell models containing mechanism-based fluorescent protein toxicity pathway reporters to quantitatively assess the dynamics of cellular stress response pathway activation at the single cell level using automated live cell imaging. We have systematically evaluated the application of four key adaptive stress pathway reporters for the prediction of DILI liability: SRXN1-GFP (oxidative stress), CHOP-GFP (ER stress/UPR response), p21 (p53-mediated DNA damage-related response) and ICAM1 (NF-κB-mediated inflammatory signaling). 118 FDA-labeled drugs in five human exposure relevant concentrations were evaluated for reporter activation using live cell confocal imaging. Quantitative data analysis revealed activation of single or multiple reporters by most drugs in a concentration and time dependent manner. Hierarchical clustering of time course dynamics and refined single cell analysis allowed the allusion of key events in DILI liability. Concentration response modeling was performed to calculate benchmark concentrations (BMCs). Extracted temporal dynamic parameters and BMCs were used to assess the predictive power of sub-lethal adaptive stress pathway activation. Although cellular adaptive responses were activated by non-DILI and severe-DILI compounds alike, dynamic behavior and lower BMCs of pathway activation were sufficiently distinct between these compound classes. The high-level detailed temporal- and concentration-dependent evaluation of the dynamics of adaptive stress pathway activation adds to the overall understanding and prediction of drug-induced liver liabilities.

**Electronic supplementary material:**

The online version of this article (10.1007/s00204-018-2178-z) contains supplementary material, which is available to authorized users.

## Introduction

Despite major efforts to understand and predict drug-induced liver injury (DILI), unpredicted liver failure upon drug use remains an important adverse drug reaction both in the clinic and during drug development (Raschi and de Ponti 2015). To be able to improve prediction of DILI liabilities from new molecular entities integration of mechanistic information is essential. Gene expression analysis has contributed significantly to our understanding of DILI (Laifenfeld et al. 2014; Raschi and De Ponti 2017). This has led to the identification of specific signaling pathways that are activated during DILI and are possibly predictive for chemical-induced liver injury. Key among these are classic stress responses activated to maintain cellular homeostasis, including the oxidative stress response, the endoplasmic reticulum (ER) stress response, the DNA damage response (Laifenfeld et al. 2014) and the TNF signaling pathway (Chen et al. 2015). We have established a panel of fluorescent protein reporter liver cell lines based on transgenomics GFP tagging, that capture each of these four pathways using Srxn1, CHOP, p21 and ICAM1 as quantitative biomarkers (Wink et al. 2017).

For the oxidative stress response pathway we have established a Srxn1-GFP reporter (Wink et al. 2017). The activation of transcription factor Nrf2 is dependent on the redox sensor Kelch-like ECH-associated protein 1 (Keap1), and induces expression of a large panel of proteins involved in the antioxidant responses including the small redox protein (Srxn1) (Jeong et al. 2012; Espinosa-Diez et al. 2015). Srxn1 is best-characterized for its ATP-dependent reduction of the hyperoxidized form of peroxiredoxin (Jeong et al. 2012). Srxn1 activity seems essential for peroxiredoxin function and protection against oxidative damage (Jeong et al. 2012). Furthermore, de-glutathionylation of s-glutathionylated cysteins by Srxn1 is essential to maintain proper phosphatase function (Findlay et al. 2006). In vivo studies show that toxicant-induced upregulation of Srxn1 via Nrf2 activation in the liver is vital for protection against fulminant oxidative stress and subsequent organ failure (Bae et al. 2011, 2012; Wu et al. 2012).

To monitor unfolded protein response (UPR) or ER stress we have established a CHOP-GFP reporter (Wink et al. 2017). The UPR is a protective response upon the accumulation of untranslated proteins in the ER. UPR activates three classical signaling pathways through PKR-like ER kinase (PERK), activating transcription factor 6 (ATF6), and inositol-requiring enzyme 1 (Ire1). Activation of PERK leads to arrest of protein translation but permits translation of activating transcription factor 4 (ATF4) which on its turn targets expression of ER function-related proteins including transcription factor C/EBP homologous protein (CHOP). Also activation of the Ire1 and ATF6 ER stress pathways can induce CHOP expression (Li et al. 2014). CHOP modulates the expression of many genes, including apoptotic machinery components. CHOP expression seems linked to the progression of liver injury and CHOP expression is induced by various cytotoxic drugs (Foufelle and Fromenty 2016).

To monitor DNA damage we established a fluorescent protein reporter for the p53 downstream target gene p21 (Wink et al. 2017). The cellular protective response upon DNA damage induces cell cycle arrest and subsequent senescence (Wink et al. 2014). p53 can be activated upon DNA damage as well as other cellular stresses, and induces expression of many target genes, including p21 (El-Deiry 2016). The best-characterized function of p21 is its effective inhibition of cyclin-dependent kinases (CDK), which halts the progression of the cell cycle. Localization of p21 has been found both in the nucleus and the cytoplasm (Ćmielová and Ŕezáčová 2011). In the liver, in vivo studies show upregulated p21 nuclear expression upon drug exposure, mostly via p53 activation (Bandi et al. 2011; Yafune et al. 2013).

Finally, a fluorescent reporter for ICAM1 allows monitoring the cytokine-mediated activation of NF-κB signaling (Tian et al. 2005). Inflammation is involved in drug-induced liver injury and repair (Chen et al. 2015). The pro-inflammatory cytokine TNFα has a central role in drug-induced liver injury (Shaw et al. 2009). Activation of the TNF receptor causes nuclear translocation of the transcription factor nuclear factor κB (NF-κB) driving the expression of various pro-inflammatory molecules, including intercellular adhesion molecule 1 (ICAM1) (Brenner et al. 2015). ICAM1 is expressed at the membrane of TNFα-activated hepatocytes, facilitating the adherence of leukocytes (Rahman and Fazal 2009). ICAM1 is widely used as a marker for inflammation and ICAM1 expression is also increased upon inflammation in the liver (Hoque et al. 2013).

Given the central role of the above stress response pathways in liver injury, our objective was to evaluate the application of our panel of target gene GFP reporter cell lines that represent these four major adaptive stress response pathways to predict DILI liability. Previously, we demonstrated that the GFP reporters allow the quantification of the chemical-induced stress responses similar to primary human hepatocytes (Wink et al. 2017). Here, we systematically determined the application of our Srxn1-GFP, CHOP-GFP, p21-GFP and ICAM1-GFP reporters for the assessment of DILI using a set of 118 FDA-labeled drugs with defined DILI drug label classification. The concentration- and time-dependent GFP responses were determined in association with general markers of cytotoxicity. In this study, we established quantitative information of the dynamic adaptive stress response activation by all 118 drugs allowing detailed mode-of-action assessment. We used the temporal dynamic stress response activation data together with concentration response modeling for the prediction of DILI outcome.

## Materials and methods

### Raw data

All image-derived data has been made publicly available at the EMBL-EBI BioStudies repository, under accession number S-BSST117.

### Cell culture

Human hepatoma HepG2 cells were acquired from ATCC (clone HB8065). HepG2 Srxn1, DDIT3 (CHOP), CDKN1A (p21) and ICAM1 BAC GFP reporter cell lines were previously established and characterized (Wink et al. 2017). HepG2 BAC GFP reporters were maintained and exposed to drugs in DMEM high glucose supplemented with 10% (v/v) FBS, 25 U/mL penicillin and 25 µg/mL streptomycin. The cell lines were used between passage 5 and 25. For live cell imaging, the cells were seeded in Greiner black µ-clear 384 wells plates, at 8000 cells per well.

### Reagents

All reference compound chemicals were acquired from Sigma–Aldrich and freshly dissolved in DMSO; except for metformin (PBS), acetaminophen and phenobarbital (all DMEM). TNFα was acquired from R&D Systems (Abingdon, UK). All 118 DILI compounds were a kind gift from the Dr. Weida Tong, NCTR-FDA (Chen et al. 2011). All compounds were maintained as 500-fold stock such that final treatments did not exceed 0.2% v/v DMSO.

### Microscopy

Accumulation of GFP levels, propodium iodide (PI) and Hoechst staining was monitored using a Nikon TiE2000 confocal laser microscope (lasers 540, 488 and 408 nm), equipped with an automated stage and perfect focus system at 37 °C with humidified atmosphere and 5% CO_2_/air mixture. All imaging was similar as previously described^5^. Each 384-well plate contained one reporter cell line, which was exposed to all the compounds used in the screen at one certain concentration (1, 5, 10, 50 or 100 C_max_); for each concentration at least two replicates were imaged per reporter cell line. For the ICAM1-GFP reporter experiments, cells were first exposed for 8 h to compound only; next, TNFα was added to all wells, up to a final concentration of 10 ng/mL. Directly after TNFα treatment the live cell imaging was started.

### Image analysis of fluorescent protein reporter activity

Quantitative image analysis was performed with CellProfiler version 2.1.1 (Kamentsky et al. 2011) with an in house developed CellProfiler module implementing the watershed masked algorithm for segmentation (Yan and Verbeek 2012; Wink et al. 2017). Image analysis results were stored as HDF5 files. Data analysis, quality control and graphics were performed using the in house developed R package h5CellProfiler (manuscript in preparation). For each reporter hourly intensity levels of the GFP signal, the nuclear Hoechst33342 intensity levels and at 24 h the PI staining were measured at the single cell level. In addition, cell numbers, nuclei size and cell motility were measured.

### Data analysis

GFP intensity cell population means were calculated. In addition, for each plate the cell population mean GFP intensity of the DMSO treated cells was calculated to determine background control values. Per plate, the single cells that had values above the 2*X* mean, 3*X* mean were counted resulting in GFP positive fraction measures. For ICAM1, the background control values consisted of DMSO conditions treated with TNFα, and the single cells with values above, as well as below background values were counted. Due to the non-symmetric distribution of ICAM1 cell population GFP intensities, the interquartile range (IQR) was used to count the number of cells 1.5, 2 and 3 times above and below the TNFα IQR control values (Supplemental Fig. 1).

To account for PI background staining noise the PI segmentations were masked by a 2 pixel dilated nuclei. The area of these nuclei and the PI objects were divided to obtain a PI/nuclei ratio. These ratios were filtered to be at least 10% of the cell size and following this procedure each cell was either flagged as alive or dead in the final time point of the 24 live imaging session. PI positive fraction was normalized to DMSO (or TNFα for ICAM1) by subtracting the control PI positive fractions.

Linear regression was applied with respect to time to quantify treatment effects on a plate-to-plate basis to quantify cell speed, nuclear size, Hoechst nuclear intensity and cell numbers. The slope coefficient mean over all plates was used to obtain a compound-concentration specific summary statistic. All summary features were scaled between 0 and 1 with the formula (*x* − *x*_min_replicate_)/(*x*_max_replicate_ − *x*_min_replicate_), with the exception of (1) the cell count features which were scaled between 0 and 1 by calculating cell fractions and (2) the ICAM1 intensity features which were scaled between − 1 and 1 to account for up or down regulation of the TNFα-induced ICAM1-pathway (Supplemental Fig. 1).

### Concentration response data transformation and benchmark concentration (BMC) modeling

The maximum values over time of the scaled intensity levels and positive GFP fractions were selected for the concentration response curves. These values were fit to a four parameter log-logistic model using the drc package (Ritz et al. 2015). BMC values were calculated as the concentration at which + 0.25 (and − 0.25 for ICAM1) absolute increase from the initial response values occurred (Fig. 5a).

The replicate means of the maximum over time features were calculated for each compound-concentration preceding unsupervised hierarchical clustering.

For the time course analysis natural cubic splines with 8° of freedom were fit after which 24 discrete equidistant time points were selected to calculate per-time point replicate means. The time course hierarchical clustering was performed by first calculating Manhattan based distances between all time-course vectors. The mean Manhattan based distances over all reporters was used as inputs for the Ward based clustering. This ensured appropriate clustering of also the temporal dynamics.

### Data representation

All HCI data representations were generated or modified with Illustrator CS6, Fiji, ggplot2 (Wickham 2009), the ‘aheatmap’ function of the NMF package (Gaujoux and Seoighe 2010).

### Severe vs non-severe DILI prediction with support vector machine

FDA DILI-annotation was used as ‘ground truth’ with non-DILI (*n* = 16), less-severe DILI (*n* = 36) and ambiguous DILI (*n* = 12) grouped as ‘non-severe DILI’ and severe-DILI (*n* = 54) as ‘severe DILI’ resulting in a two-classification problem. Features were obtained by time dynamic feature extraction of time courses with functional data analysis using the in house developed R-package ‘celloscillate’ and the BMC and C-max normalized BMC values. Feature selection and SVM model tuning was performed in a 200 times iterative process with randomly selected 80/20—equal class distributed training/test set procedure. The training phase included a first feature selection step using a Kolmogorov–Smirnov test for equal distributions between the two classes followed by pair-wise correlation filter step (> 0.8 or < − 08). The second step in the training phase consisted of the SVM model tuning with ten repeats of 10-fold cross-validation. The test phase on 20% of the compounds was performed using the selected features and tuned SVM model. Reported prediction results are the average of the 200 test-set runs; the ROC distribution of the test-runs were defined. Hierarchical clustering of the 20 selected features corresponded to the features selected > 150 times through the 200 iterations.

### Gene expression analysis

CEL files were downloaded from the Open TG-GATEs database: “Toxicogenomics Project and Toxicogenomics Informatics Project under CC Attribution-Share Alike 2.1 Japan” https://dbarchive.biosciencedbc.jp/en/open-tggates/desc.html and analyzed as described previously (Wink et al. 2017).

### Statistics

For all reporters and concentrations three independent biological replicate imaging experiments were performed, of which upon selection of cell viability of untreated control conditions, at least two replicates were used for the quantification of reporter activity and further statistical analysis. For statistical significance of all time courses first linear interpolation was applied for each separate time course using the ‘approx’ function from the R-stats package to obtain 100 equal discretized time points for each replicate. The high number of linear interpolations was required to retain the original noise in the time course data. Following this step, a one-way ANOVA for functional data method was applied using the ‘anova.onefactor’ function of the R-package fda.usc to determine significant difference in time-curves compared to DMSO for Srxn1/CHOP/p21 or TNFα for ICAM1. Multiple testing correction was applied using the fdr-method (Benjamini and Hochberg). Srxn1/CHOP/p21 were assessed for significant upregulation and ICAM1 for significant down- or up-regulation.

For the log-BMC values a linear model with the BMC and C-max as explanatory variables was fit as null-model. The null-model was compared in an anova to a model containing DILI-class as additional additive explanatory variable. The models were compared in an anova for significant effect of DILI-class.

For the C-max normalized BMC a Welch two-sample *t* test was performed between the severe and non-severe DILI groups.

## Results

### High content adaptive stress response screen with DILI compounds

**Fig. 1:**
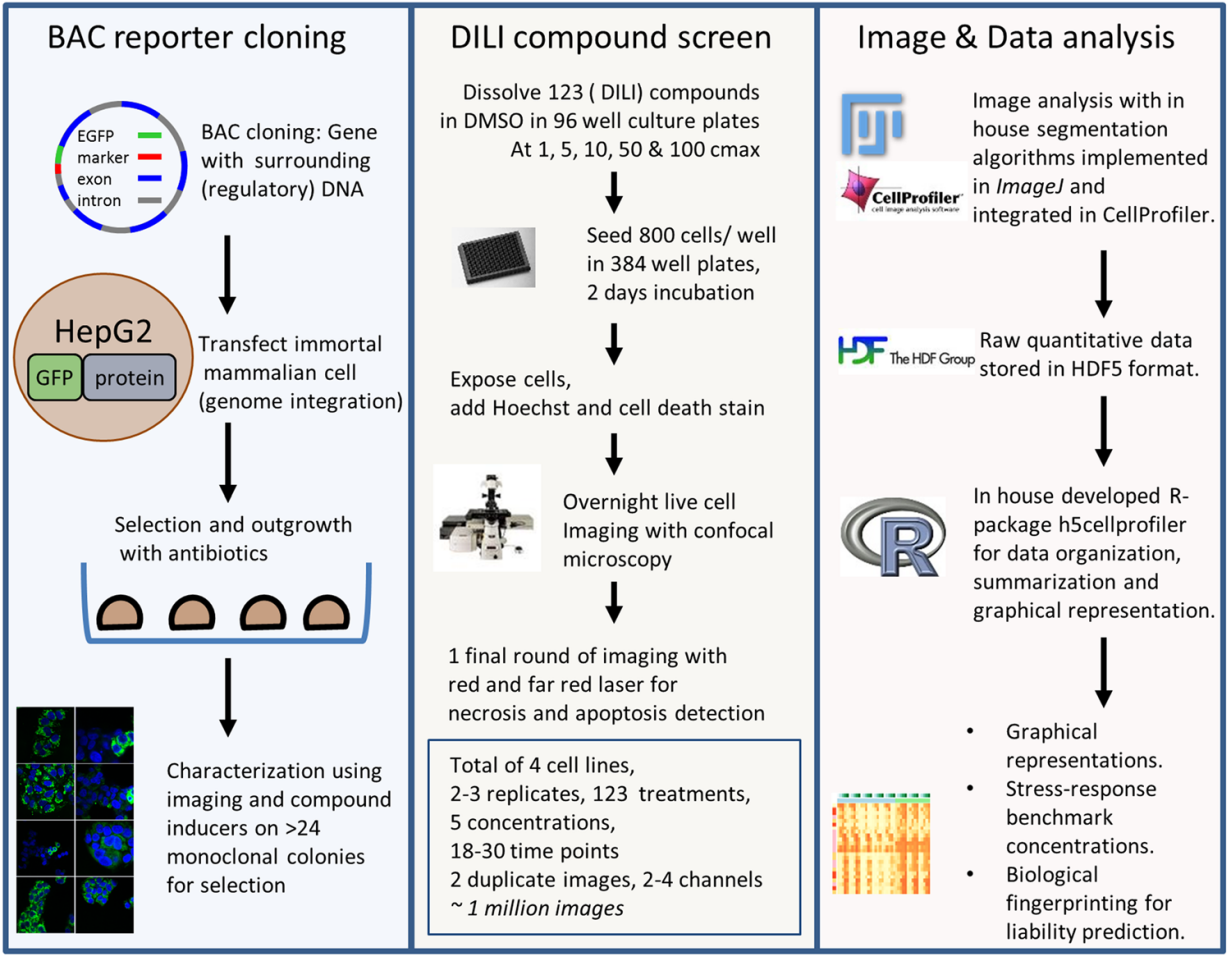
BAC cloning, BAC reporter DILI screen and analysis pipeline. Left panel BAC cloning technology is used to maintain endogenously regulated reporter protein levels and regulation. Monoclonal reporter selection from a high number of clones to ensure endogenous response to positive control stimuli and suitability of reporter for imaging. Middle panel high content live cell screen of 123 compounds at 1, 5, 10, 50 and 100 C-max at 2 or 3 replicates. Right panel image and data analysis is performed with CellProfiler/Fiji and R, respectively. In-house tools were developed in CellProfiler and R to assist in the quality and analysis of the large data output

**Fig. 2:**
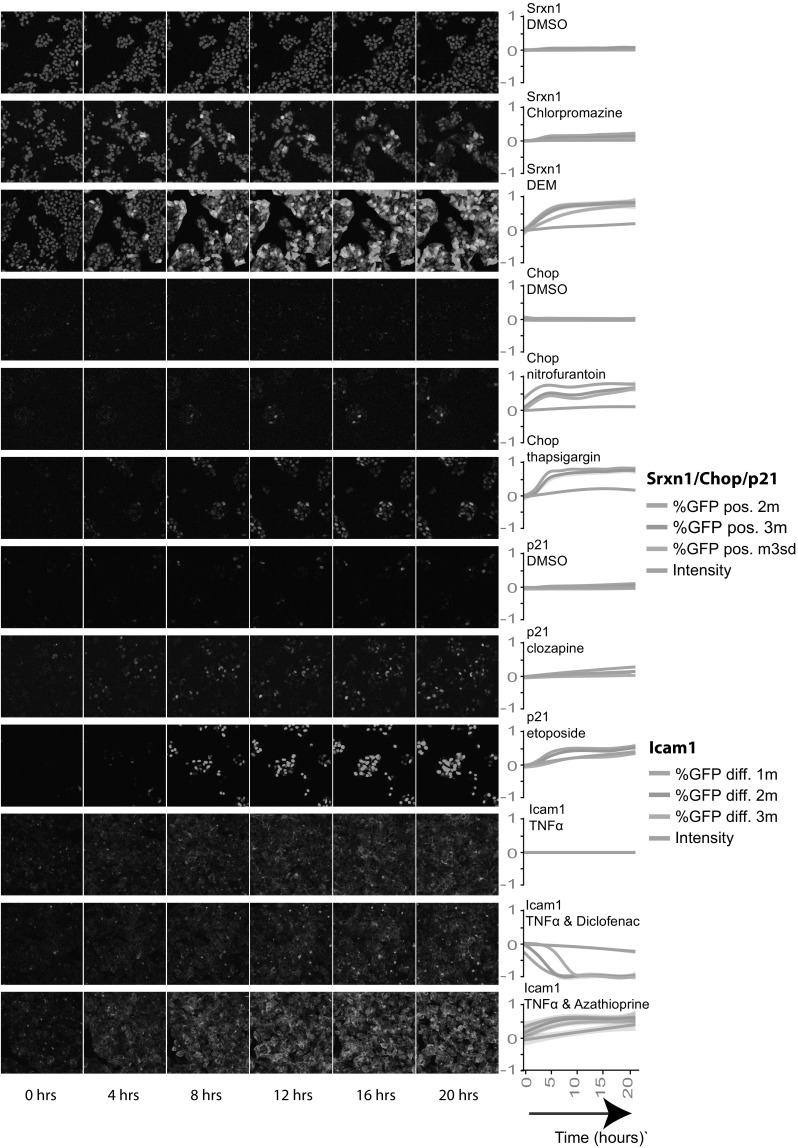
Dynamics of Srxn1-GFP, CHOP-GFP, p21-GFP and ICAM1-GFP reporter activation. Left panel time lapse images of the reporters exemplifying the importance of single cell analysis which allows fine tuning sensitivity vs dynamic range of BAC-reporter read-out. Right panel quantification of GFP signal of the Srxn1-GFP, p21-GFP and CHOP-GFP reporters from a control, a weak reporter-activating compound and a strong reporter-activating compound. For ICAM1-GFP the TNFα control was accompanied by a compound which induced the TNFα induced response and by a compound which reduced the TNFα response. Intensity, %GFP positive 2 m, %GFP positive 3 m and %GFP positive m3sd are shown for Srxn1-GFP, CHOP-GFP and p21-GFP. For ICAM1-GFP, Intensity, %GFP diff. 1 m, %GFP diff. 2 m and %GFP diff. 3 m are shown

To assess the application of adaptive stress response pathway activation for assessment of adverse drug reactions, we focused on DILI. For the assessment of DILI liabilities we screened 123 compounds, of which 118 with known DILI liabilities (Fig. 1; Table 1). As an adaptive stress response read-out, HepG2 BAC-GFP reporter cell lines for oxidative stress (Srxn1-GFP), ER-stress (CHOP-GFP), DNA damage (p21-GFP) and inflammatory cytokine signaling stress (ICAM1-GFP) were used [(Wink et al. 2017) and see Suppl. Figure 2]. Stress response activation following DILI drug exposure was monitored with live cell confocal microscopy for a period of 24 h. The time-resolved single cell data was quantified using an established image analysis pipeline (Wink et al. 2017). For labeling DILI compounds we used the FDA DILI labeling, which labels drugs either as no-DILI-concern, ambiguous DILI-concern, less-severe DILI concern or most-severe-DILI-concern (Chen et al. 2016). Most-severe-DILI-concern drugs are highly associated with DILI and represent multiple specialist verified cases of DILI. Less-severe-DILI-concern drugs represent few verified cases of DILI. If drugs are suspected to cause most- or less-severe-DILI-concern, but the presented cases cannot be validated by experts, drugs received the ambiguous DILI-concern label. No-DILI-concern drugs are on the market for decades and have never been associated with DILI. To separate out clear examples of DILI, we made two classes: ‘non-severe-DILI’ and ‘severe-DILI’, where the most-severe-DILI-concern drugs comprised the ‘severe-DILI’ group and all others are in the ‘non-severe-DILI’ group. In addition, we included FDA labeling in eight separate classes of hepatotoxicity ranging from no hepatotoxicity to fatal hepatotoxicity (Table 1). The screen also included reference compounds including negative controls (DMSO and medium) and positive controls (i.e. DNA damage inducers, soft electrophilic alkylating agents and ER stress inducers) (see Table 1; Fig. 2).

**Table 1:** Test compound set

Compound	C_max_ (μ)	Abb.	DILI con.	Seventy	Hepa.	C_max_ ref.	Metab
Acarbose	0.15	ACA	Most-DC	Sev.	8	FDA	NM
Acetaminophen	139	APAP	Most-DC	Sev.	5	Xu/O’Brien	YES
Adefovir	0.085	ADV	Less-DC	Non-sev.	2	Dailymed	NM
Allopunnol	13.81	ALLO	Most-DC	Sev.	8	FDA	NM
Altretamine	3.76	ALM	Amb.-DC	Non-sev.	2	FDA	YES
Amiodarone	0.807	AMIO	Most-DC	Sev.	8	Xu/O’Brien/Khet.	NM
Amoxicillin	22.3	AX	Less-DC	Non-sev.	5	FDA	NM
Azathioprme	0.34	AZA	Most-DC	Sev.	5	FDA	YES
Benzbromarone	4.339	BB	Most-DC	Sev.	8	FDA	YES
Betaine	940	BET	No-DC	Non-sev.	1	O’Brien	NM
Bicalutamide	1.97	BAT	Most-DC	Sev.	8	FDA	YES
Bosentan	7.4	BOS	Most-DC	Sev.	7	Dawson/Gustaf.	YES
Bromfenac	17.96	BFC	Most-DC	Sev.	8	FDA	NA
Buspirone	0.016	BUS	Amb.-DC	Non-sev.	3	FDA	YES
Busulfan	0.277	BU	Most-DC	Sev.	8	FDA	VES
Captopril	8.882	CPL	Less-DC	Non-sev.	7	FDA	VES
Carbamazepine	50.79	CBZ	Most-DC	Sev.	7	Dailymed	NM
Chloramphenicol	46.36	CAMP	No-DC	Non-sev.	1	Ogutu	NM
Chlormezanone	10.59	CMZ	Most-DC	Sev.	8	FDA	NA
Chlorpromazine	0.94	CPZ	Less-DC	Non-sev.	2	Xu	YES
Chlorpropamide	130.1	CHL	Less-DC	Non-sev.	2	FDA	NM
Cimetidine	11.89	CMT	Less-DC	Non-sev.	2	Regenthal	YES
Ciprofloxacin	6.58	CIPX	Most-DC	Sev.	7	FDA	NM
Clofibrate	470	CLO	Less-DC	Non-sev.	3	O’Brien	YES
Clotrimazole	0.087	CTZ	Less-DC	Non-sev.	3	FDA	NM
Clozapine	2.44	CLZ	Most-DC	Sev.	5	Regenthal	YES
Colchicine	0.016	CLC	Amb.-DC	Non-sev.	6	FDA	YES
Cyclosporin A	0.2	CSA	Most-DC	Sev.	NA	Dailymed	YES
Dacarbazine	20.64	DTIC	Most-DC	Sev.	6	FDA	NM
Danazol	0.109	DNZ	Most-DC	Sev.	8	FDA	NM
Dantrolene	7.9	DAN	Most-DC	Sev.	8	FDA	YES
Dexamethasone	0.224	DXS	Amb.-DC	Non-sev.	3	FDA	NM
Dextromethorphan hbr	0.022	DXM	No-DC	Non-sev.	NA	Xu	NA
Diclofenac	10.1	DCF	Most-DC	Sev.	8	Gustaf./Regen.	YES
Didanosine	9.83	DDL	Most-DC	Sev.	8	FDA	NM
Diethylmaleate		DEM	Control	Na	NA	NA	NA
Digoxin	0.003	DIG	No-DC	Non-sev.	1	FDA	YES
Diltiazem	0.356	DTZ	Most-DC	Sev.	4	Pat el	YES
Disulfiram	5.4	DIS	Most-DC	Sev.	8	FDA	YES
Dmso	0.2	DM SO	Control	Na	NA	NA	NA
Edrophonium	60.2	EDR	No-DC	Non-sev.	1	FDA	NA
Enalapril	0.4	ENP	Less-DC	Non-sev.	7	FDA	YES
Entacapone	3.93	ECP	Less-DC	Non-sev.	1	Dailymed	YES
Epinephrine	0.002	EPI	No-DC	Non-sev.	1	FDA	NA
Erythromycin	11	ERVC	Most-DC	Sev.	5	FDA	NM
Ethambutol	24.47	EMB	Most-DC	Sev.	8	FDA	NM
Etodolac	68.49	ELAC	Most-DC	Sev.	8	FDA	YES
Etoposide		ETO	Control	Na	NA	NA	YES
Famotidine	0.308	FAM	Less-DC	Non-sev.	3	FDA	YES
Fenofibrate	4.1	FF	Less-DC	Non-sev.	3	FDA	NM
Fenoprofen	58.2	FPF	Most-DC	Sev.	8	FDA	YES
Fialuridine	1	FIAU	Most-DC	Sev.	8	O’Brien	NA
Fluoxetine	0.05	FLX	Less-DC	Non-sev.	3	Regenthal	YES
Flurbiprofen	57.32	FBP	Amb.-DC	Non-sev.	3	FDA	YES
Folic acid	0.043	FAM	No-DC	Non-sev.	1	FDA	YES
Furosemide	3.29	FUR	Amb.-DC	Non-sev.	2	FDA	YES
Ganciclovir	4.62	GOV	Amb.-DC	Non-sev.	7	FDA	YES
Glimepiride	1.12	GLP	Less-DC	Non-sev.	7	FDA	NM
Griseofulvin	4.54	GF	Most-DC	Sev.	8	FDA	YES
Haloperidol	0.005	HDL	Less-DC	Non-sev.	5	FDA	YES
Hydroxyzine	0.27	HVZ	No-DC	Non-sev.	1	FDA	NM
Imipramine	0.29	IM	Less-DC	Non-sev.	3	FDA	YES
Indomethacin	5.59	IMN	Most-DC	Sev.	8	FDA	YES
Iboniazid	76.56	INH	Most-DC	Sev.	8	Xu/Regen./Daw.	YES
Isoproterenol	2.02	IPR	No-DC	Non-sev.	1	Gustafsson	NA
Kanamycin	60.1	KM	No-DC	Non-sev.	1	Xu	NA
Ketoconazole	6.59	KTZ	Most-DC	Sev.	8	Khetani	NM
Ketorolac	3.53	KTL	Less-DC	Non-sev.	3	FDA	NM
Labetalol	2.68	LABE	Most-DC	Sev.	8	FDA	VES
Maprotiline	0.18	MPT	Amb.-DC	Non-sev.	5	FDA	NA
Mebendazole	0.13	MBZ	Less-DC	Non-sev.	3	FDA	NM
Meclizine	0 026	MCZ	No-DC	Non-sev.	1	FDA	NM
Mercaptopurine	0.48	6MP	Most-DC	Sev.	8	FDA	YES
Metformin	7.78	MF	Less-DC	Non-sev.	1	Regenthal	YES
Methimazole	2.62	MTZ	Most-DC	Sev.	8	FDA	VES
Methotrexate	0.77	MXT	Most-DC	Sev.	3	Regenthal	NM
Methyldopa	18.94	MD	Most-DC	Sev.	8	FDA	YES
Metoprolol	0.56	MTPL	Less-DC	Non-sev.	5	FDA	YES
Mexiletine	3.83	MXT	Most-DC	Sev.	3	FDA	YES
Moxisylyte	0.16	MOX	Most-DC	Sev.	8	FDA	NA
Naproxen	0.2	NPX	Less-DC	Non-sev.	3	Regenthal	VES
Nefazodone	3.95	NFZ	Most-DC	Sev.	8	Gustaf./Regen ./Daw.	YES
Neomycin	0.44	NEO	No-DC	Non-sev.	1	Hu: in vitro dose	NM
Nifedipine	0.43	NFP	Less-DC	Non-sev.	3	Wagner	YES
Nimesulide	21.082	NMS	Most-DC	Sev.	8	FDA	YES
Nitrofurantoin	6	NTF	Most-DC	Sev.	8	FDA	YES
Nizatidine	4	NIZ	Less-DC	Non-sev.	5	FDA	YES
Ofloxacin	9.96	OFX	Less-DC	Non-sev.	3	Dailymed	NM
Omeprazole	4.7	OMZ	Less-DC	Non-sev.	4	Dailymed	YES
Oxytetracycline	3.26	OTC	Amb.-DC	Non-sev.	2	FDA	NA
Paroxetine	0 061	PXT	Less-DC	Non-sev.	8	FDA	YES
Perhexiline	2.16	PHX	Most-DC	Sev.	8	Xu	NA
Phenobarbital	145.5	PBT	Less-DC	Non-sev.	3	Schmidt	NM
Phenytoin	21.72	PT	Most-DC	Sev.	8	Xu et al	YES
Pioghtazone	2.946	PGZ	Less-DC	Non-sev.	3	Xu/O’Brien	NM
Prednisolone	0.68	PRD	Less-DC	Non-sev.	3	FDA	NM
Primaquine	0 615	PQ	No-DC	Non-sev.	1	Xu	NM
Primidone	4.67	PRI	No-DC	Non-sev.	1	FDA	YES
Procychdine	0 404	PCD	No-DC	Non-sev.	1	FDA	NA
Propranolol	0 201	PPL	Amb.-DC	Non-sev.	3	FDA	YES
Propylthiouracil	9.1	PTU	Most-DC	Sev.	8	FDA	YES
Ranitidine	1.79	RNT	Less-DC	Non-sev.	5	FDA	YES
Ribavirin	2.61	RBV	Amb.-DC	Non-sev.	7	FDA	YES
Rifampicin	15	RFP	Most-DC	Sev.	NA	Dailymed	YES
Simvastatin	0 082	SVN	Less-DC	Non-sev.	3	FDA	YES
Succinylcholine	137.74	SUCCS	No-DC	Non-sev.	1	FDA	NA
Sulindac	31.985	SUL	Most-DC	Sev.	8	FDA	YES
Tacrolimus	0 037	TAC	Less-DC	Non-sev.	5	Dailymed	YES
Tamoxifen	0 162	TMX	Most-DC	Sev.	8	Xu	YES
Terbinafine	4	TRB	Most-DC	Sev.	8	FDA	NM
Thapsigargin		TG	Control	Na	NA	NA	NA
Thiondazine	0.55	TDZ	Less-DC	Non-sev.	5	Ravic	YES
Ticlopidine	8 075	TPD	Most-DC	Sev.	4	FDA	YES
Tnfα		TNF	Control	Na	NA	NA	NA
Tolbutamide	233.03	TOLB	Amb.-DC	Non-sev.	2	FDA	NM
Tolcapone	21.99	TC	Most-DC	Sev.	8	Dailymed	YES
Trazodone	5.056	TZ	Less-DC	Non-sev.	5	FDA	YES
Troghtazone	6.39	TRG	Most-DC	Sev.	8	Xu/Khetani	YES
Verapamil	0.5	VRP	Less-DC	Non-sev.	3	Regenthal	NM
Warfarin	4.86	WAR	Less-DC	Non-sev.	5	FDA	NM
Ximelagatran	0.3	XML	Most-DC	Sev.	8	Keisu	NA
Zafirlukast	1.21	ZFL	Most-DC	Sev.	8	FDA	YES
Zimehdine	0 267	ZMI	Most-DC	Sev.	8	Gustafsson	NA

Alphabetically sorted list of screened compounds in this study including their C-max values, abbreviations, DILI-concern labeling, severity class, hepatotoxicity class (1 = no hepatotoxicity, 2 = cholestasis/steatohepatitis, 3 = liver aminotransferases increase, 4 = hyperbilirubumenia, 5 = jaundice, 6 = liver necrosis, 7 = acute liver failure, 8 = fatal hepatotoxicity), C-max reference (Schmidt et al. 1986; Regenthal et al. 1999; Ogutu et al. 2002; Ravic et al. 2004; O’Brien et al. 2006; Xu et al. 2008; Andersson and Keisu 2010; Nidhi et al. 2011; Patel et al. 2011; Dawson et al. 2012; Hu and Coates 2013; Khetani et al. 2013; Gustafsson et al. 2014) and metabolic potential based on the livertox.nih.gov database (*YES* compound is metabolized, *NM* compound is not metabolized, *NA* not available in the database)

### Single cell analysis of adaptive stress response activation

All reporters were exposed to five concentrations: 1, 5, 10, 50 and 100 C-max followed by automated live cell imaging and multi-parametric image analysis (Fig. 1); C-max values were obtained either from FDA or from literature (see Table 1) (Schmidt et al. 1986; Regenthal et al. 1999; Ogutu et al. 2002; Ravic et al. 2004; O’Brien et al. 2006; Xu et al. 2008; Andersson and Keisu 2010; Nidhi et al. 2011; Patel et al. 2011; Dawson et al. 2012; Hu and Coates 2013; Khetani et al. 2013; Gustafsson et al. 2014). For all images single cell analysis was performed to extract a diverse set of quantitative data, including GFP reporter activity, cell number and markers of cytotoxicity (Suppl. Figure 1). Srxn1-GFP, p21-GFP and CHOP-GFP reporter single cell data was used to derive quantitative data for different determinants of reporter activity: intensity and fraction of cells with GFP intensity levels above control values. Fraction of cells with GFP intensity were divided in three types: two times or three times the mean of the control values (i.e. %GFP positive 2 m and %GFP positive 3 m, respectively); and the control mean plus three times the standard deviation of the mean (%GFP positive m3sd). All ICAM1-GFP reporter drug exposures were primed with TNFα exposure; likewise, ICAM1-GFP shows a gradual increase over 24 h time period in the vehicle control. As a consequence, drug treatment can lead to an enhancement or inhibition of the TNFα-induced ICAM1-GFP response. ICAM1-GFP fractions were calculated as the difference between up- and downregulated fractions (%GFP diff. 2 m). Systemic evaluation of these descriptors for the least and strongest responding compound for each individual reporter allowed fine tuning of the sensitivity versus the dynamic range (Fig. 2). For example, based on the Srxn1-GFP intensity over the single cell population chlorpromazine would not have been defined as positive in the Srxn1-GFP reporter cell line, because only in a small proportion of cells that contain a higher level of Srxn1-GFP the signal was detected. Yet, the %GFP positive 2 m and %GFP positive 3 m were more sensitive descriptors that also allowed evaluation of the time course dynamics for chlorpromazine. Similar observations were made for nitrofurantoin and clozapine for the CHOP-GFP and p21-GFP reporters, respectively. However, for strong inducers of oxidative stress (diethylmaleate; DEM), ER-stress (thapsigargin) and DNA damage (etoposide), GFP mean intensity already allowed detection of the reporter responses, while %GFP positive 2 m caused an early saturation, thereby lowering the information value of the temporal dynamics. Further, diclofenac (DCLF) and azathioprine (AZA) showed inhibiting and enhancing modulatory effects on TNFα-induced ICAM1-GFP, respectively.

### DILI compounds demonstrate specific stress response reporter activation dynamics

**Fig. 3:**
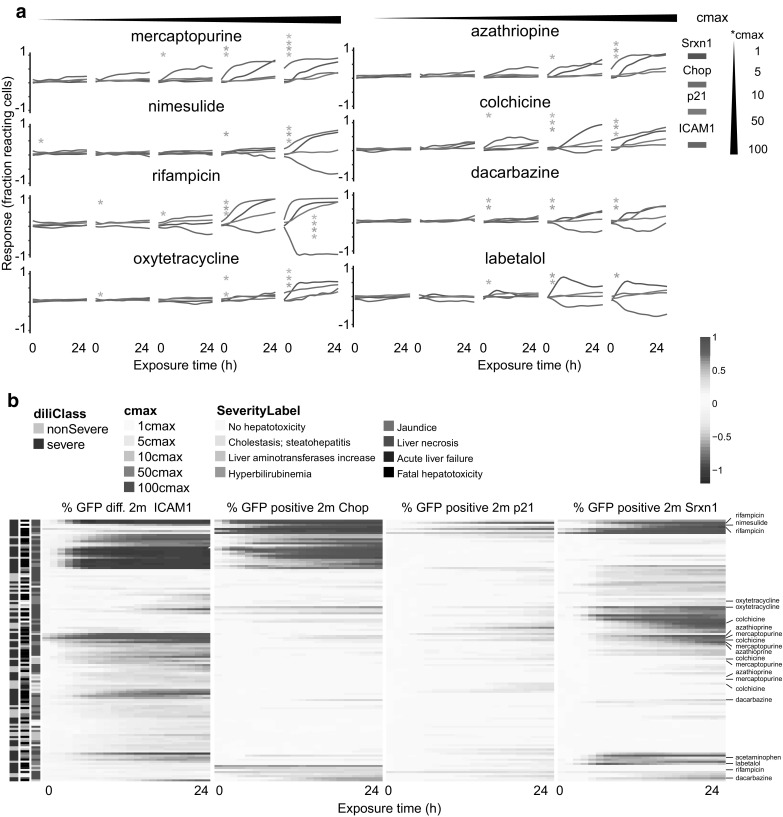
Time dynamics of a subset of the screened drugs. **a** GFP responses over time of %GFP positive 2 m of Srxn1-GFP, CHOP-GFP and p21-GFP and of GFP_dif.2 m of ICAM1-GFP. Statistics are performed as described in the material and methods section and represent *< 0.01 with the corresponding color to dissect between the different reporter lines. **b** Zoom of hierarchical clustering of time dynamic responses. Red is an upregulation and blue is a downregulation. On the left the severe/non-severe (purple) and the hepatotoxicity class (grey) labeling are indicated as well as the C-max values (green). (Color figure online)

For the evaluation of the reporter activation for the entire compound screen %GFP positive 2 m was selected as the most sensitive initial readout. The %GFP positive 2 m time courses were used to calculate the mean of the replicates for Srxn1-GFP, CHOP-GFP, p21-GFP and ICAM1-GFP reporter responses for all compounds (Fig. 3a and Suppl. Figure 3). Some compounds showed a response in all four reporters. Thus, methyldopa (MD) increased Srxn1-GFP, CHOP-GFP and p21-GFP activity and inhibited the ICAM1-GFP response. Mercaptopurine (6MP) increased all four reporters. The data also allowed discrimination of specific time dynamics of stress activation. Thus, for nimesulide (NMS), rifampicin (RFP) and oxytetracycline (OXY) an initial CHOP-GFP response at 100× C-max and a delayed Srxn1-GFP response was observed. In contrast, for azathioprine (AZA), colchicine (CLC) and dacarbazine (DTIC) a Srxn1-GFP response was later followed by CHOP-GFP, suggesting ER-stress as the primary mode-of-action (Fig. 3a). Hierarchical clustering of the time courses from all 118 compounds representing the reporter activities from all BAC-GFP reporter cell lines demonstrated a large cluster with considerable modulation of stress response reporter activity (Fig. 3b and Suppl. Figure 4). This cluster showed an overrepresentation of severe-DILI compounds as well as more severe classes of hepatotoxicity (liver necrosis, acute liver failure and fatal hepatotoxicity). Sub-clusters with activation of all four reporters were established, with ICAM1-GFP either up- or downregulated. p21-GFP did show few responses and did not contribute much to the DILI compound clustering, in overall agreement with anticipated overall lack of DNA damage of marketed drugs, excluding anticancer drugs. The time response clearly demonstrated the dynamics of the various stress response programs and allowed discrimination between primary stress types and subsequent secondary responses. Strikingly, suppression by DILI compounds of the cytokine-induced ICAM1-GFP expression was highly correlated with activation of the CHOP-GFP reporter, which in a few cases was co-occurring with Srxn1-GFP activation.

### Concentration response analysis reveals strong clustering of severe DILI compounds

**Fig. 4:**
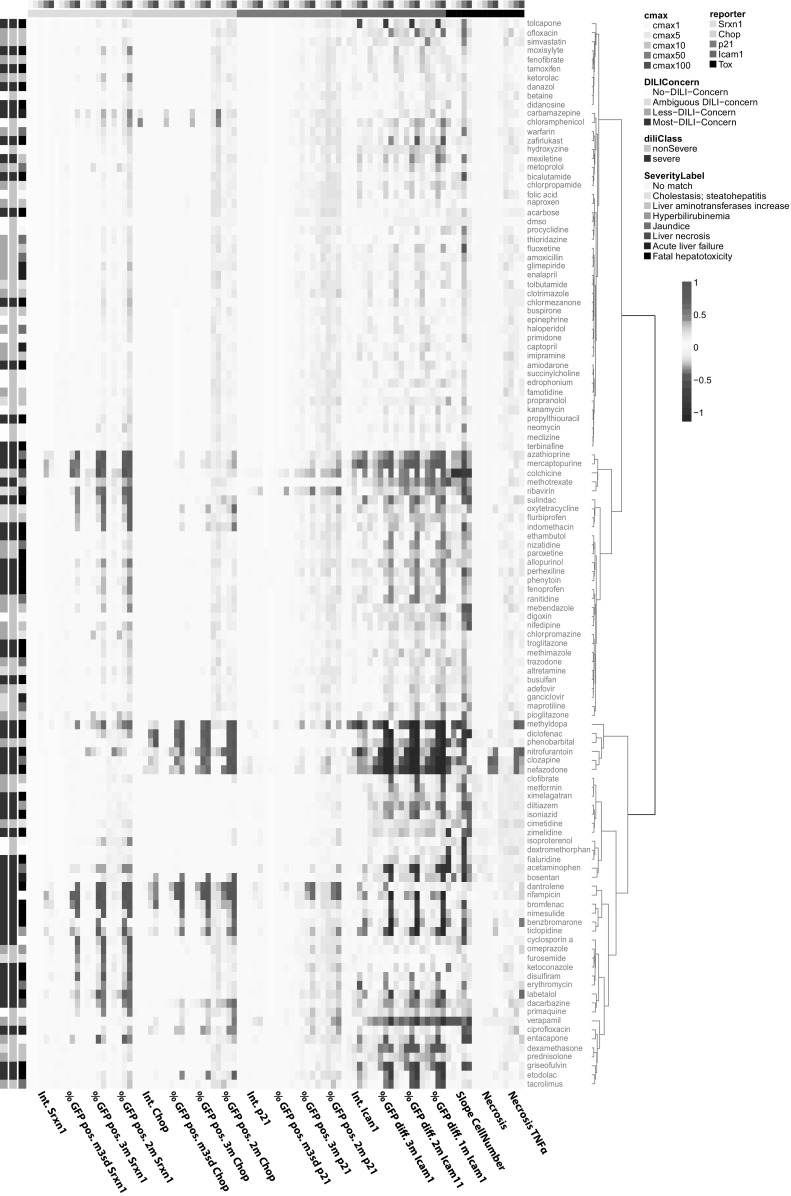
Hierarchical clustering of peak GFP response in time Hierarchical clustering of responses of intensity and count related GFP responses in dose response fashion. Cell death measurements cell number and PI staining are in the right bar. On the left side DILI labeling is depicted in three bars: severe/non-severe, DILI-concern labeling and hepatotoxicity class labeling. Compound names are colored based on the unsupervised clustering. (Color figure online)

Next, we summarized time course data by extracting the time point at which the reporter expression reached a peak response using the various quantitative GFP reporter activity descriptors as well as cytotoxicity measurements. Hierarchical clustering revealed a stress response reporter active group divided over three sub-groups and one large non activated group (Fig. 4). For the active group one sub-group was marked by an increase in Srxn1-GFP, CHOP-GFP and (for some compounds) p21-GFP in combination with a decrease in ICAM1-GFP. A second sub-group was characterized by a strong increase in CHOP-GFP and cytotoxicity as well as a strong decrease in ICAM1-GFP. For the third sub-group both Srxn1-GFP and ICAM1-GFP reporters showed a clear increase. Most of the severe-DILI compounds were present in one of the active sub-groups. The point-of-departure (PoD) varied between reporters. For example mercaptopurine showed a relatively strong activation of both SRXN1-GFP and ICAM-GFP, yet, the PoD for ICAM-GFP response was at a lower C-max than for the onset the SRXN1-GFP response. In contrast, ketoconazole showed no Srxn1-response at the intensity feature level but only at the more sensitive %GFP positive marker starting earliest at 50 C-max as the primary and only stress-type. Thus, the current high content data analysis revealed the value of measuring quantitative adaptive stress responses for the different DILI classes with a clear distinction in primary stress responses for individual DILI compounds.

### Benchmark concentrations definition reveals low PoD for Srxn1-GFP and CHOP-GFP activation in the severe DILI group

**Fig. 5:**
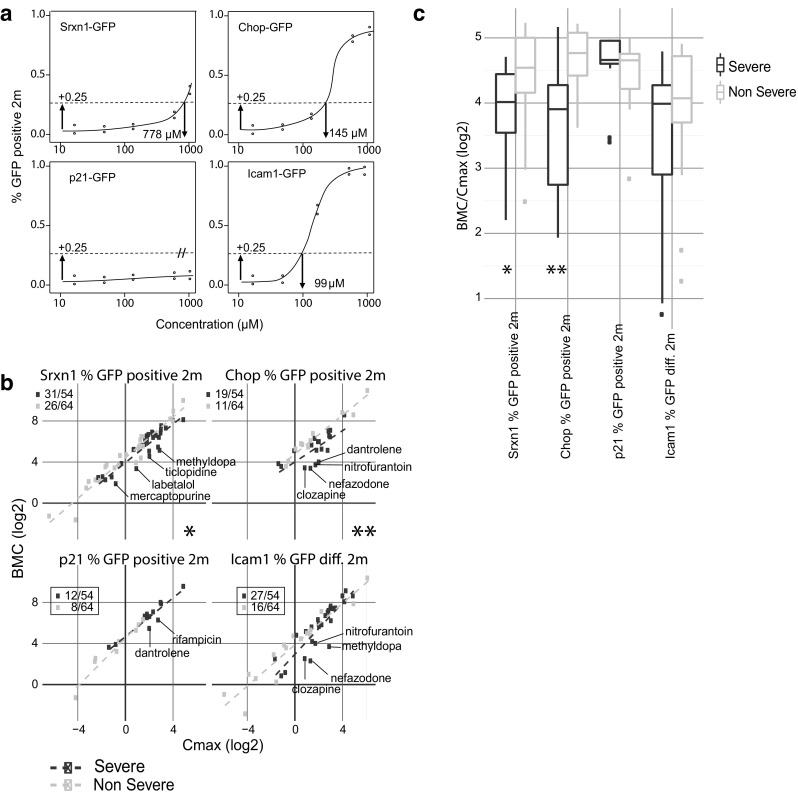
Benchmark concentration (BMC) versus the absolute C-max values per reporter. **a** Explanation of how we extract BMC from the fitted dose ranges from different GFP reporters. **b** For each reporter the absolute BMC (y-axis) is plotted against the absolute C-max (x-axis), each dot represents a compound which reached the 0.25 threshold. Purple indicates a severe DILI drug, Light blue indicates a non-severe drug; * = *p* < 0.05 and ** = *p* < 0.01. **c** The BMC is divided by the absolute C-max value per compound and represented in the severe/non-severe DILI classes and per reporter; * = *p* < 0.05 and ** = *p* < 0.01. (Color figure online)

Based on the concentration response curves extracted from the peak response in %GFP positive 2 m (Srxn1-GFP, CHOP-GFP and p21-GFP) and %GFP diff. 2 m (ICAM1-GFP) we defined the benchmark concentration (BMC; defined as at least 25% of the cells that reach the two times average threshold of the control values: see Fig. 5a). This BMC can function as an indicator for the PoD for further risk assessment modeling. The C-max values for the 118 DILI compounds covered a large concentration range from 1.7 nM to 0.94 mM. Therefore, we plotted the BMC against the absolute C-max value (Fig. 5b) and we divided the BMC by the absolute C-max (Fig. 5c). We observed a lower BMC in Srxn1-GFP, CHOP-GFP and ICAM1-GFP for severe DILI compounds compared to non-severe DILI compounds. This indicates that severe DILI compounds have a lower PoD and are, therefore, more potent to activate adaptive stress response pathways.

### Stress response reporter activity SVM classification and prediction of DILI liability

**Fig. 6:**
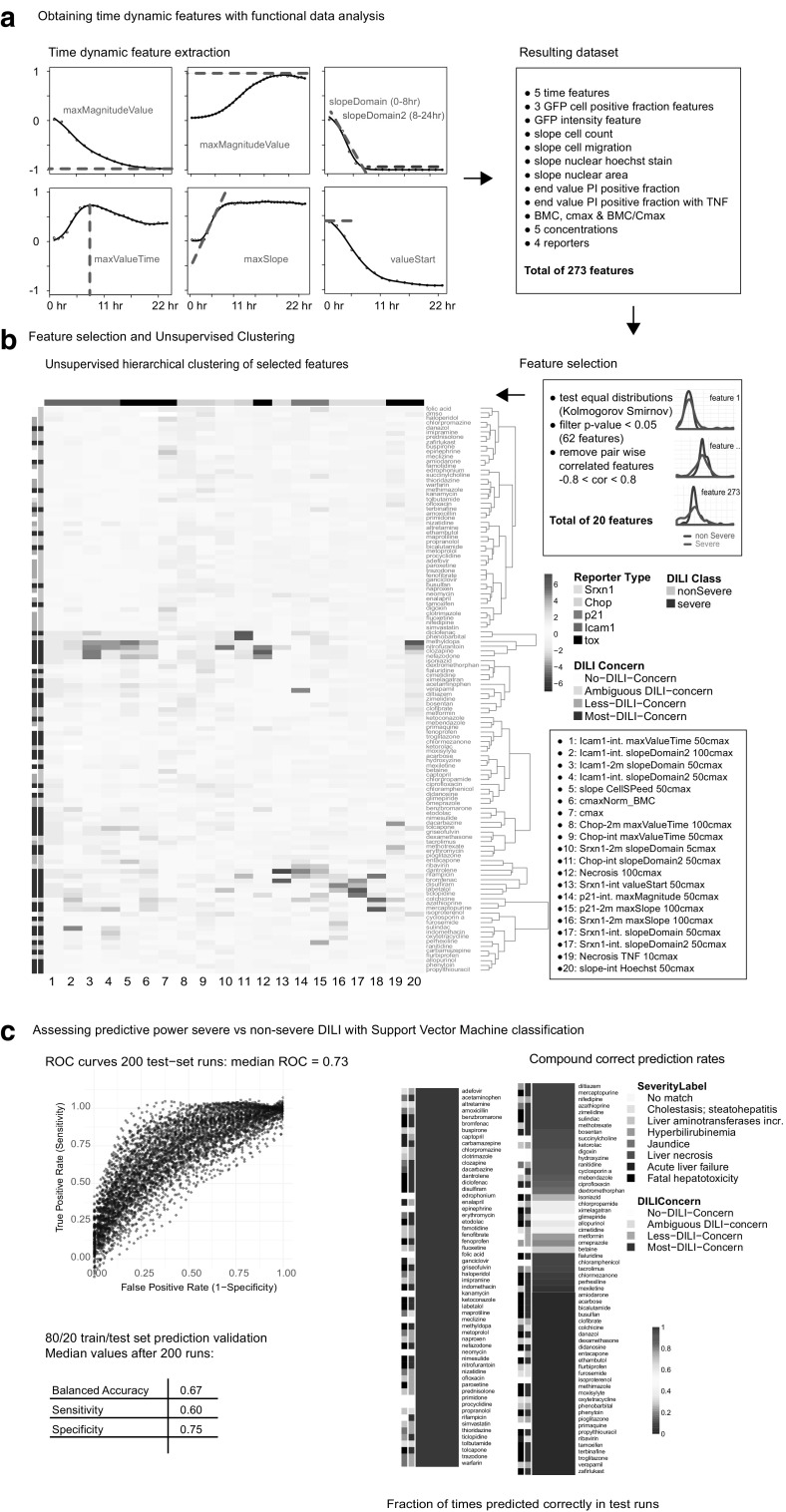
Prediction of severe versus non-severe DILI groups. **a** Explanation of temporal feature extraction. **b** Feature selection by Kolmogorov Smirnov test and pairwise correlation filter. Followed by unsupervised hierarchical clustering of the most often selected features from the 200 feature selection iterations. **c** ROC curves from 200 test data runs and average prediction results (left); Fraction of correct prediction for each drug

Next, we extracted the time dynamic features and BMC values for all 118 DILI compounds for the different reporters culminating in 273 different features. Machine learning was applied to asses temporal stress pathway activation and concentration—response relations for predictive power and feature relevance. Feature selection and support vector machine (SVM) model tuning were performed over 200 iterations of random training/test dataset sampling (Suppl. Figure 1). The features selected more than 150 times in the 200 iterations were subjected to unsupervised hierarchical clustering (Fig. 6b). Interestingly, this set included a diverse set of features, encompassing all reporters, BMC, C-max and also cytotoxicity features including cell death with TNFα at 10 C-max, cell death at 100 C-max and cell speed at 50 C-max. Early and late slope features from the reporters seem to be preferred over the max magnitude values. The resulting clustering with these features showed three dominant sub-groups with enriched severe DILI compounds (Fig. 6b).

The 200 independent test-set prediction validations with the tuned SVM model resulted in an average ROC of 0.73 (Fig. 6c, left panel) and a sensitivity of 0.60 and specificity of 0.75 with ‘positive’ being the severe-DILI group. Over the 200 runs the correct prediction rates for each compound was calculated (Fig. 6c, right panel). 88 DILI compounds were consistently either predicted correctly or predicted falsely. A smaller set of 30 compounds have some uncertainty as to being predicted correctly. No enrichment for DILI-class can be seen for these prediction rates.

## Discussion

Here, we systematically evaluated the application of a panel of four key adaptive stress response fluorescent protein reporters in high content high throughput screening as a method for DILI liability assessment. We anticipate that adaptive stress pathway activation respond well before the onset of overt toxicity, thus ensuring increased sensitivity as well as integration of detailed quantitative mechanistic information in chemical safety assessment. We monitored four downstream adaptive stress response pathways represented by Srnx1 (Nrf2 oxidative stress response), CHOP (ER-stress/UPR response), p21 (p53 dependent DNA damage-related signaling) and ICAM1 (NFκB-mediated pro-inflammatory cytokine signaling). Systematic image analysis revealed specific activation of Srxn1-GFP and CHOP-GFP by severe DILI versus non-severe DILI inducing drugs. A subset of dynamic features across all cell reporters allowed the classification of severe versus non-severe DILI classes with a sensitivity of 60% and a specificity of 75%. We demonstrate the application of advanced dynamic high content imaging data of stress response signaling can be integrated with informatics approaches for DILI inducing liability assessment of candidate drugs. This mechanism-based assessment is a fast and transparent approach to integrate mode-of-action information in prioritizing lead compounds early in the drug development cycle.

Our adaptive stress response reporters provide detailed information on the adverse mode-of-action of different drugs. We observed three different clusters of DILI inducing drugs with clear reporter activity: (1) a cluster with mild Srxn1-GFP and ICAM1-GFP activation (including azathioprine, mercaptopurine and colchicine); (2) a cluster with strong ICAM1-GFP suppression and CHOP-GFP activation (including nefazodone, clozapine and nitrofurantoin); and, (3) a cluster with mild ICAM1-GFP suppression and activation of Srxn1-GFP and/or CHOP-GFP (including diclofenac, dantrolene, bromfenac and benzbromarone). Overall, Srxn1-GFP responses were often observed in cooperation with the activation of CHOP-GFP and/or ICAM1-GFP reporter activity. This suggests that the Nrf2 activation is a primary cause which also disturbs other systems as protein folding or inflammation or that it follows a secondary effect after cellular stress induction. Since we also captured the time dependent adaptive stress activation dynamics, we were able to make a distinction between these two possibilities which both occurred (see Fig. 3 and Suppl. Figures 3 and 4). Interestingly, a very strong decrease in ICAM1-GFP activation coincided with a strong increase in CHOP-GFP activation in a similar concentration response, but no or minor activation of Srxn1-GFP. This is indicative for a specific mode-of-action of these DILI inducing compounds and suggest a relationship between ER-stress induction and suppression of TNFα-mediated NFkB activation and ICAM1 expression. Previously, we demonstrated such a mutual relationship for diclofenac and showed that inhibition of CHOP induction prevented the diclofenac/TNFα-mediated cell death (Fredriksson et al. 2014). For two other drugs in this cluster, nefazodone and clozapine, such a dual modulation of ER-stress and NFκB signaling was demonstrated before (Cosgrove et al. 2009; Lauressergues et al. 2012; Abdel-Wahab and Metwally 2015; Ren et al. 2016). Finally, we observed that a strong enhancement of TNFα-induced ICAM1-GFP activation by DILI inducing compounds was often accompanied with an increase in Srxn1-GFP as well as a decrease in cell number. Since oxidative stress can also lead to NFkB activation, there is a likely synergistic effect for the enhancement of TNFα-mediated NFκB activation. In general, no major increase of p21-GFP activation was observed for most compounds, this is a comforting observation and reflects the genuine exclusion of genotoxic effect of drugs during drug development.

Our systematic screening approach allowed defining of benchmark concentrations for all DILI inducing drugs and the respective reporters. For Srxn1-GFP, CHOP-GFP and ICAM1-GFP we observed a lower ratio of the BMC/C_max_ for severe DILI compared to non-severe DILI inducing drugs, irrespective of the of C_max_ levels. This was especially pronounced in the Srxn1-GFP and CHOP-GFP reporters, indicating that the safe therapeutic window for severe DILI compounds is limited. Therefore, since CHOP is an important adaptation-adversity-switch in ER stress signaling, activation of CHOP may then contribute to liver injury (Yamaguchi and Wang 2004). Since Srxn1-GFP contributes to adaptation, compounds that primarily activate the Nrf2 pathway to a limited extend may still be considered safe (Osburn and Kensler 2009).

The screen was performed in a time-resolved live single cell setting. To date, toxicity screening efforts using high content imaging have mostly focused on single time point fluorescent dyes or anti-bodies (Garside et al. 2014) with several real-time based toxicity screening efforts (Kim et al. 2012). However, the use of dyes and anti-bodies brings additional noise to already very noisy systems as fixation and anti-body binding are likely additional sources of variability; this is not an issue using our reporter models. With the use of our reporter cell lines biological signaling can be visualized with a high time resolution to more accurately pinpoint the primary mode-of-action in relation to cellular stress. Time course signaling data also greatly benefits computational modeling efforts as these require detailed time and dose response dynamics, this is only feasible using live cell imaging data. Furthermore, we were able to extract features based on these time and dose response dynamics. These features were used in support vector machine approach to assess possible differences in cellular adaptive signaling between less- severe and severe DILI. Due to the limited number of no-DILI compounds (*n* = 16) and the total of 118 compounds tested we had obtained a limited set of observations for building highly accurate predictive models. However, by combining the less-Severe DILI and ambiguous DILI cases together with the no-DILI compounds we were still able to show significant predictivity with an independent subset of our data not used in the SVM tuning process. A ‘predictive toxicogenomics space (PTGS)’ method has recently been defined to determine sensitivity and specificity in classification of known DILI drugs (Kohonen et al. 2017). Although in this study, a different DILI labeling strategy was used, we observed a comparable sensitivity using our limited set of stress response reporters. Interestingly, some of our reporters are reflected by the transcription factors that determine the toxicogenomics-based classifier in the PTGS approach. We anticipate that the discrepancy in the specificity is likely due to the difference in DILI negative labeling approach. We anticipate that the further development of an ever increasing reporter imaging database containing detailed signaling based features linked to chemical exposure, i.e. compound specific biological fingerprints, will ultimately aid in the safety evaluation and early (DILI) prediction of new drugs and chemicals.

We performed the screen using the HepG2 cell line. Although HepG2 has several advantages for in vitro screening (unlimited lifespan, cheap, easy to culture), the major limitation is their lack of metabolic capacity. Interestingly, several compounds that involve biotransformation-dependent toxicity demonstrated activation of an Srxn1-GFP oxidative stress response (e.g. acetaminophen and sulindac). To test whether there is a concordance between the HepG2 BAC-GFP reporters and the transcript levels in primary human hepatocytes we used the TG-GATES dataset to calculate the correlation of the activity of the reporters genes between TG-GATES and the current BAC-GFP HepG2 DILI screen (see Suppl. Figure 5). This indicates a significant correlation of Srxn1 responses at the transcript levels in primary human hepatocyte and protein expression of BAC-GFP reporters in HepG2 cells, also suggestive for a minor role for drug metabolism in the onset of DILI compound-induced stress response activation. Interestingly, for various compounds we did observe induction of stress response pathways that could not be observed in primary human hepatocytes (boxed area, Suppl. Figure 5), thus indicating increased sensitivity of the BAC-GFP reporters for some compounds. Previously, we optimized a HepG2 3D spheroid protocol to enhance liver like properties and to enable chronic exposures (Ramaiahgari et al. 2014). In future research, we can test whether this would increase prediction of DILI drugs.

In conclusion, we have shown that BAC-GFP reporter cell lines are a sensitive tool to provide detailed mechanistic information regarding the adaptive stress response activation in a broad compound screening setting using high-content live single cell imaging. Such detailed insights into the perturbations of signaling pathways after chemical exposure provides key information for safety assessment and predictive purposes. We anticipate that our BAC-GFP reporter platform will contribute to the early pre-clinical screening for DILI liabilities and possibly also other chemical safety assessment paradigms.

## Electronic supplementary material

Below is the link to the electronic supplementary material.

Supplemental Fig. 1: Data analysis workflow. The features in red are displayed in the figures of the results section (TIF 742 KB)

Supplemental Fig. 2: ICAM1-GFP characterization. a) Time lapse images with and without TNFα. b) Comparison BAC-GFP ICAM1-GFP and wild type HepG2 with western blotting after induction with TNFα and stained with ICAM1, GFP and tubulin antibodies. c) Time lapse images of ICAM1-GFP after knock down of caspase 8 and NF-κB subunit RelA and TNFα exposure (EPS 25851 KB)

Supplemental Fig. 3: Time course graphs of all treatments and concentrations. For all compounds the reporter activity is shown for %GFP positive 2m (Srxn1-GFP, CHOP-GFP and p21-GFP) and %GFP diff. 2m (ICAM1-GFP). Statistics were performed as described in the material and methods section: * = p<0.01 (EPS 4556 KB)

Supplemental Fig. 4 Hierarchical clustering of time course summarized in a heatmap. Red depicts an upregulation and blue a downregulation. On the left the severe/non-severe (purple) and the hepatotoxicity class (grey) labeling are indicated as well as the C-max values (green) (EPS 10384 KB)

Supplemental Fig. 5: Correlation of TG GATES primary human hepatocytes transcript levels versus BAC-HepG2 Srxn1-GFP (blue), CHOP-GFP (green) and p21-GFP (red). BAC-GFP reporter values are plotted against the TG-GATES fold changes. Boxed cloud depicts BAC-GFP reporter activated compounds only. Srxn1: correlation of 0.36 with 61 df and a p-value of 0.003, Chop: correlation of 0.22 with 61 df and a p-value of 0.085, p21: correlation of 0.19 with 61 df and a p-value of 0.1266 (EPS 2161 KB)

Supplemental Fig. 6: DILI labeling. Severity-label and DILI-class as published (Chen et al. 2016). DILI-class was defined to create a two-class classification system as described in the main text. All Most-DILI-Concern was assigned to the severe class, the remaining DILI compounds to the nonSevere class (EPS 610 KB)
