# Supplementary figures and images for: Dynamic imaging of adaptive stress response pathway activation for prediction of drug induced liver injury
Source: Arch Toxicol. 2018 Mar 3;92(5):1797–814. doi: 10.1007/s00204-018-2178-z (PMC5962642; doi:10.1007/s00204-018-2178-z)

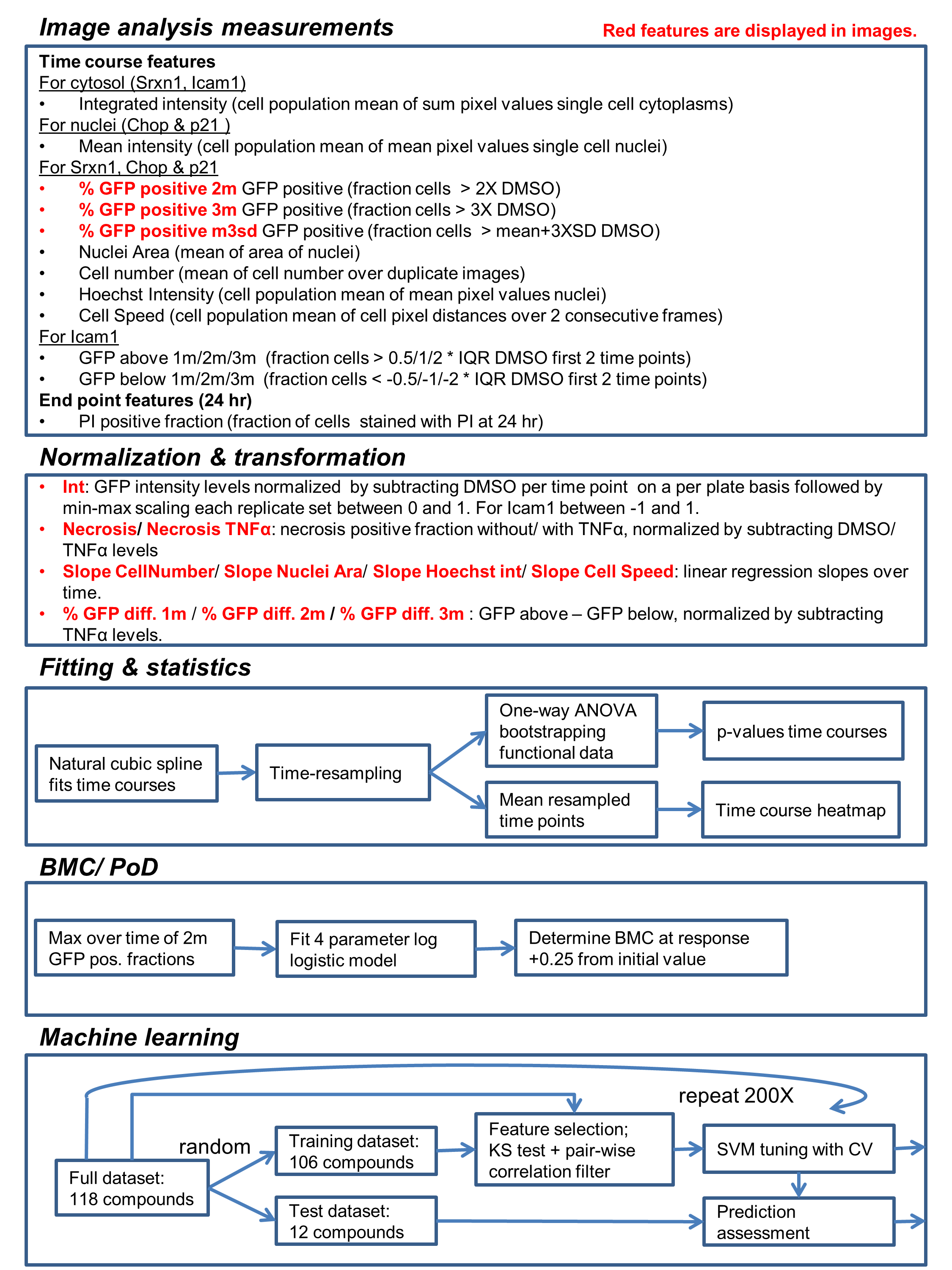

Supplement: Supplementary file 1 — Supplemental Fig. 1: Data analysis workflow. The features in red are displayed in the figures of the results section (TIF 742 KB) [file 204_2018_2178_MOESM1_ESM.tif]
